# Functional validation of novel compound heterozygous variants in B3GAT3 resulting in severe osteopenia and fractures: expanding the disease phenotype

**DOI:** 10.1186/s12881-016-0344-9

**Published:** 2016-11-21

**Authors:** Florian Job, Shuji Mizumoto, Laurie Smith, Natario Couser, Ashley Brazil, Howard Saal, Melanie Patterson, Margaret I. Gibson, Sarah Soden, Neil Miller, Isabelle Thiffault, Carol Saunders, Shuhei Yamada, Katrin Hoffmann, Kazuyuki Sugahara, Emily Farrow

**Affiliations:** 1 Institute for Human Genetics and Molecular Biology, Martin Luther University Halle-Wittenberg, Magdeburger Str. 2, 06112 Halle (Saale), Germany; 2 Department of Pathobiochemistry, Faculty of Pharmacy, Meijo University, 150 Yagotoyama, Tempaku-ku, Nagoya, Aichi 468-8503 Japan; 3 University of North Carolina School of Medicine, Division of Pediatric Genetics and Metabolism, Department of Pediatrics, Raleigh, NC USA; 4 Division of Human Genetics, Cincinnati Children’s Hospital Medical Center, Cincinnati, OH 45229 USA; 5 Department of Pathology, Children’s Mercy Hospitals and Clinics, Kansas City, MO USA; 6 Department of Pediatrics, Children’s Mercy Hospitals and Clinics, Kansas City, MO USA; 7 Department of Medical Informatics, Children’s Mercy Hospitals and Clinics, Kansas City, MO USA; 8 The Laboratory of Proteoglycan Signaling and Therapeutics, Graduate School of Life Science, Faculty of Advanced Life Science, Hokkaido University, Sapporo, 001-0021 Japan; 9 Center for Pediatric Genomic Medicine, Children’s Mercy Hospitals and Clinics, 2420 Pershing, Suite 100, Kansas City, MO USA

**Keywords:** B3GAT3, Glycosaminoglycan, Glucuronyltransferase-I, Linkeropathy, Proteoglycan

## Abstract

**Background:**

A new disease class of syndromes, described as linkeropathies, which are derived from defects in the glycosaminoglycan-linker region as well as glycosaminoglycan-side chains of proteoglycans is increasingly being recognized as a cause of human disease. Proteoglycans are an essential component of the extracellular matrix. Defects in the enzymatic process of proteoglycan synthesis broadly occur due to the incorrect addition of side chains. Previously, homozygous missense variants within the *B3GAT3* gene encoding beta 1,3 glucuronyltransferase 3(GlcAT-I) responsible for the biosynthesis of glycosaminoglycans have been described in 7 individuals.

**Case presentation:**

In this study, a 4-year-old patient with a severe phenotype of osteoporosis, hypotonia, joint laxity, fractures, scoliosis, biscuspid aortic valve and myopia was referred for next generation sequencing after extensive negative clinical testing. Whole exome sequencing was performed on the proband and his unaffected parents to identify the molecular basis of his disease. Sequencing revealed compound heterozygous variants in *B3GAT3*: c.1A > G (p.Met1?) and c.671 T > A (p.L224Q). Clinical and in vitro functional studies were then completed to verify the pathogenicity of the genotype and further characterize the functional basis of the patient’s disease demonstrating the patient had a decrease both in the protein level of *B3GAT3* and in the glucuronyltransferase activity when compared to control samples. Independent in vitro assessment of each variant confirmed the *B3GAT3*: c.1A > G (p.Met1?) variant is functionally null and the c.671 T > A (p.L224Q) missense variant has significantly reduced glucuronyltransferase activity (~3% of control).

**Conclusions:**

This is the first report of a patient with compound heterozygosity for a null variant in trans with a missense in *B3GAT3* resulting in a severe phenotype, expanding both the genotypic and phenotypic spectrum of B3GAT3-related disease.

**Electronic supplementary material:**

The online version of this article (doi:10.1186/s12881-016-0344-9) contains supplementary material, which is available to authorized users.

## Background

A new class of syndromes, described as linkeropathies, which encompass defects in the glycosaminoglycan (GAG)-linker region as well as GAG-side chains of proteoglycans is increasingly being recognized as the cause of human disease [1, 2]. Proteoglycans are an essential component of extracellular matrix, particularly in cartilage, bone and the cardiovascular system [3–5]. They consist of a core protein produced in the endoplasmic reticulum, with a linker region that is added in the endoplasmic reticulum and Golgi apparatus [6]. The addition of disaccharide units, epimerisation and sulfation by various glycosyltransferases, sulfotransferases, and epimerases result in the biosynthesis of GAG side chains including heparan, chondroitin, and dermatan sulfates of proteoglycans [1, 7, 8]. Defects in any step of the enzymatic process of proteoglycan synthesis cause a decrease in mature GAGs [1, 2, 7]. Specifically, *B3GAT3*, beta-1,3-glucuronyltransferase 3 (glucuronosyltransferase I, GlcAT-I), encodes a glycosyltransferase that functions to add the final glucuronic acid in the linker region tetrasaccharide, xylose-galactose-galactose-glucuronic acid, which is attached to specific serine residues on core proteins [9, 10]. Variants in *B3GAT3* (Larsen Syndrome, OMIM #245600), as well as *B3GALT6* (Spondyloepimetapyseal Dysplasia with Join Laxity, OMIM # 271640), *B4GALT7* (Ehlers-Danlos syndrome with short stature and lim anomalies, OMIM #130070), and *XYLT1* (Desbuquois dysplasia-2, OMIM #615777) have been associated with linkeropathies, which are broadly associated with joint laxity, fractures, and cardiac malformations [11–14]. However, these are a relatively new class of disorders, therefore it is unlikely the phenotypic spectrum has been fully recognized. Specifically, for *B3GAT3*, only 7 individuals, all with homozygous missense variants have been described ranging in phenotypic severity from mild to severe, with all patients having congenital heart defects, joint hypermobility, and fractures [11, 15–17].

Herein we describe the first patient with compound heterozygosity for a null variant in trans with a missense in *B3GAT3* identified as the cause of disease and provide functional characterization.

## Materials and methods (See Additional file 1: Supplemental Information for Detailed Methods)

### Whole exome sequencing

Samples were prepared for whole exome sequencing using the Nextera Rapid Capture Exome Kit (Illumina, San Diego, CA) according to manufacturer’s protocols. Sequencing was completed on an Illlumina HiSeq 2500 instrument with TruSeq v4 reagents, yielding paired end 125 nucleotide reads, with an average of 12.7 GB of data resulting in a mean 66× coverage. Gapped alignment to reference sequences (GRCh37.p5) was performed with GSNAP and the GATK and analysis completed using custom-developed software, RUNES and VIKING as previously reported [18, 19].

### Cell culture

Fibroblasts for cmh000720 utilized in the current studies were obtained when the patient was approximately 5 months old. Age- and sex- matched control fibroblasts were used for the functional assays.

### Western blotting

Twenty micrograms of fibroblast cell lysate of patient and matched control were separated on a 10% SDS-PA gel. Primary anti-B3GAT3 antibody (Abnova, Atlanta, GA) was for staining. As an additional loading control experiment, membranes were stripped and stained with anti-actin antibody (1:5000, polyclonal mouse; BD Bioscience).

### qPCR

RNA was isolated from confluent primary fibroblast cultures using Trizol (Ambion/Thermo Fisher, Grand Island, NY). *B3GAT3* mRNA levels were analyzed in primary fibroblasts by quantitative PCR (qPCR) using the ProtoScript® First Strand cDNA Synthesis Kit (NEB, Ipswich, MA). Two sets of intron spanning primers were designed for *B3GAT3* mRNA (Additional file 1: Table S1) and GAPDH was used as an endogenous control. Relative expression was determined using the 2 − ΔΔCt method [20].

### GlcAT-I assay of the recombinant wild-type and mutant, L224Q, GlcAT-I proteins

The expression vector of human GlcAT-I (wild-type), p3xFLAG-CMV8/hGlcAT-I, was used as described previously [11], and the mutant vector was constructed by overlapping extension PCR method [21]. Each vector was transiently transfected into HEK293T cells using the FuGENE HD DNA-transfection reagent (Promega, Madison, WI, USA). Three days after transfection, an aliquot of the conditioned media was individually incubated with an anti-FLAG affinity agarose resin (Wako, Osaka, Japan) at 4 °C for 4 h. To examine the expression of both recombinant enzymes, SDS-PAGE and western blotting were performed using an anti-FLAG antibody.

Glucuronyltransferase activity was examined as described previously [11]. Briefly, the enzyme-bound resin as an enzyme source, UDP-[^14^C]-GlcA (Perkin Elmer, Boston, MA, USA) as the sugar donor substrate, and Galβ1-3Galβ1-*O*-methyl (Sigma, St. Louis, MO, USA) as the sugar acceptor were utilized for the assay. The reaction mixtures were incubated at 37 °C for 20 min. The radiolabeled products were separated from UDP-[^14^C]-GlcA using anion-exchange resin, AG 1-×8 (PO_4_^2−^ form), as described previously [22]. The isolated products, [^14^C]-GlcAβ1-3Galβ1-3Galβ1-*O*-methyl, were quantified in a liquid scintillation counter (LSC-7400, Hitachi-Aloka, Tokyo, Japan).

### Comparison of the GlcAT-I activities of fibroblast homogenates

The homogenates of the fibroblasts were assayed using Galβ1-3Galβ1-*O*-methyl as an acceptor (220 nmol) and UDP-[^14^C]-GlcA as a donor substrate, and then incubated for 4 h at 30 °C. The procedures thereafter were described above.

### Cell-based ELISA

Cell-based ELISA was carried out using the protocol provided by R&D Systems Inc. (https://www.rndsystems.com/products/cell-based-elisas) with slight modifications. Briefly, fibroblasts from the patient and a control subject were cultured to determine the amount of CS. Cells were washed and treated with chondroitinase ABC at 37 °C for 30 min. After washing the chondroitinase-treated cells were fixed, incubated with the primary antibodies, a mixture of commercial anti-CS-stub antibodies (1B5, 2B6, and 3B3) (Cosmo Bio Co., LTD, Tokyo, Japan). Subsequently the cells were incubated with a secondary antibody, alkaline phosphatase-conjugated anti-mouse IgG. Finally, the cells were incubated with the substrate, *p*-nitrophenyl phosphate, and analyzed by measuring the absorbance at 405 nm with an iMark microplate absorbance reader (Bio-Rad, Hercules, CA, USA).

### Characterization of the mutation in start codon of GlcAT-I

An expression vector of the C-terminally GFP-tagged human GlcAT-I, pAcGFP-hGlcAT-I (wild-type), was constructed. To examine the effects of variants in the start codon of *GlcAT-I* on the expression of the encoded proteins, mutant vectors were constructed in frame with C-terminal GFP tag. Each vector was transiently transfected into HEK293T as described above. To examine which ATG is utilized as the initiation codon, SDS-PAGE and western blotting were performed using an anti-GFP antibody, mFX75 (Wako, Japan).

## Case presentation

The patient, cmh000720, is currently a 6-year-old male who was referred for whole exome sequencing at 4 years of age. The patient was conceived via artificial insemination and was born at an estimated 38 weeks gestation via C-section to a 32-year-old G3P2 mother and distantly related 27-year-old father (5th cousins once removed). The pregnancy was complicated by gestational diabetes that was well managed with diet. Decreased fetal movements were noted throughout pregnancy, however the results from ultrasound exams and maternal serum AFP testing were normal. At birth the patient was noted to have a week cry, hypotonia, joint hypermobility, and an undescended testicle. Apgar scores were 8 and 9 at 1 and 5 min respectively; weight was 3.1 kg (30^th^%ile) and length 50.8 cm (68^th^%ile). He was discharged home at 2 days of life. Newborn screening was reported to be normal. At a routine pediatric follow up appointment at two weeks of age the patient was hospitalized for additional evaluation due to concerns of his persistent low tone and cardiac murmur. At that time he was noted to have both a left hip dislocation and right hip subluxation, with bilateral acetabular dysplasia. Use of a Pavlik harness failed correct the hip dislocation due to profound ligamentous laxity. Echocardiogram revealed a PFO and biscupid aortic valve. The patient had inconsistent TSH levels and thus was treated with levothyroxine. An MRI was suggestive of an arachnoid cyst, but was otherwise unremarkable. At 2.5 months of age an EEG revealed abnormal sharp waves, and CSF glucose levels were found to be 38. Due to a concern for a GLUT1 deficiency, a ketogenic diet was trialed but discontinued after the patient became lethargic on the diet and repeat testing was normal. The patient has had no documented clinical seizure activity.

**Fig. 1:**
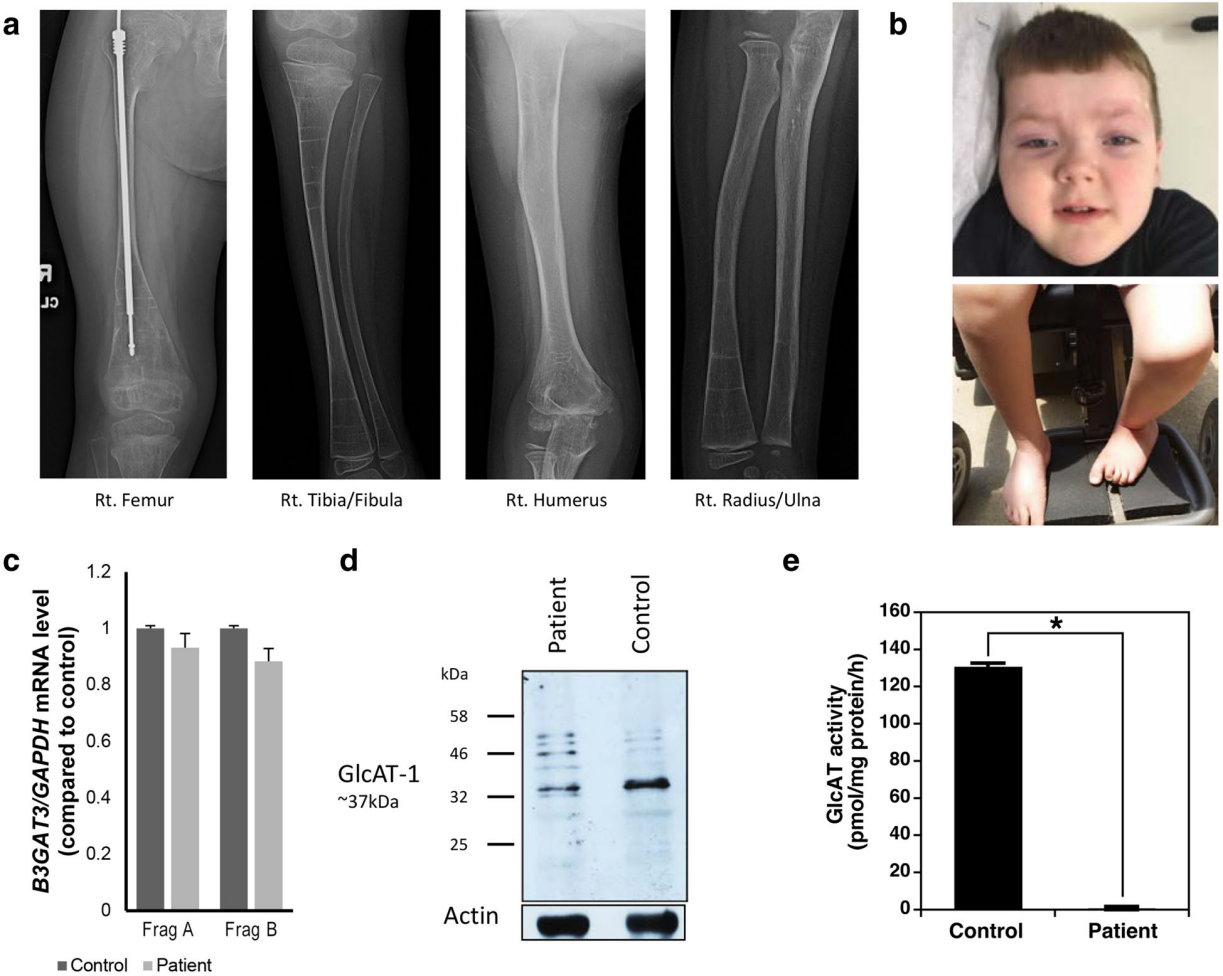
**a** X-rays revealed severely diffusely demineralized bones, along with multiple growth arrests lines. Stabilization rods are in both femurs and bilateral bowing of the tibiae and fibulae are evident (*right pictured*). **b** Cmh000720 is mildly dysmorphic with downslanting palpebral fissures (*left*), short neck, and curved lower extremities. While able to ambulate with a walker and physical assistance, he is wheelchair-bound. **c** mRNA expression of B3GAT3 in primary fibroblasts in cmh000720 was not statistically different from control using two sets of primers, whereas western blotting (**d**) showed a decrease in overall protein. **e** Glucuronyltransferase activity was determined by incorporation of [^14^C]GlcA into the Galβ1-3Galβ1-*O*-methyl. The control sample utilized an enzyme source from the fibroblasts from a healthy control subject. *P < 0.0001

In addition to his hip dysplasia, cmh000720 was also noted to have significant kyphoscoliosis and osteopenia by 4 months of age. He has had multiple fractures associated with little to no trauma including his left tibia (12 months), right femur (19 months), right tibia (20 months), left femur (2.5 years), right femur shaft (3.75 years), distal right femur (3.8 years), and left femur (4.2 years). Pamidronate infusions were trialed but an increase in fractures was noted. He has significant osteoporosis; bone density by dexa of the left forearm at 4 years of age was measured as Z score of -5.8. Spinal fusion rods were placed at 3 years of age and removed at 5 years of age. He is currently in a hard brace. Stabilization rods were also placed in both femurs (Fig. 1a).

The patient had developmental delay of gross motor milestones; currently he is able to walk with a walker and bear weight but cannot walk independently. Cognitively he is normal; he was able to say single words at 12 months and currently only has problems with articulation. At 6 years of age, in addition to the aforementioned symptoms, the patient is followed for mild aortic root and ascending aorta dilation, restrictive lung disease requiring C-PAP/BiPAP due to scoliosis and chronic obstructive pulmonary disease, bilateral hyperopia, astigmatism, amblyopia, and mild left ptosis, and hypoglycemia with illness. His weight 24.76 kg (85^th^%ile) and length 111 cm (13^th^%ile) are within normal limits, although he still receives supplemental nutrition overnight through a G-tube that was placed at 20 months of age. He is macrocephalic with an OFC of 54.8 cm (>98^th^%ile). He is only mildly dysmorphic with downslanting palpebral fissures, bluish/grey sclera, a high arched palate, short neck, arachnodactyly, and hyperextensible skin with no striae (Fig. 1b). His bone age is estimated at 4 years 6 months (>2 SD below expected for chronological age). He completed kindergarten with an IEP that includes occupational, speech and physical therapies.

The family history is notable for an older brother who was found to have a biscuspid aortic valve, after recommended screening. He has a healthy younger sister. Echocardiograms of both parents and younger sister were normal. Both parents are tall, and reportedly healthy.

## Results

Prior to whole exome sequencing, the patient had extensive biochemical and genetic testing all of which were normal or non-diagnostic. Whole exome sequencing of the patient and both parents revealed compound heterozygous variants in *B3GAT3*: a paternally inherited c.1A > G (p.Met1?) and maternally inherited c.671 T > A (p.L224Q). Subsequent Sanger sequencing of both siblings revealed that the older brother who also has a biscuspid aortic valve did not inherit either variant while the younger sister is heterozygous for the c.1A > G (p.M1?) variant (Additional file 1: Figure S1). While the c.1A > G variant has not been reported in any public database and is unique to the family in an internal database, a different variant at the same position has been reported in 2 heterozygous individuals in ExAC (2/62,938). This variant is predicted to result in loss of translation initiation and was interpreted as likely pathogenic according to current ACMGG guidelines [23].

The maternally inherited c.671 T > A (p.L224Q) variant is in exon 4 and replaces a nonpolar leucine residue with a polar glutamine residue, a moderate physicochemical difference. The leucine is conserved across species (Additional file 1: Figure S2); and is predicted to be disease causing. The variant has not been reported in any public databases and is unique to the family in an internal database. Of note, a homozygous variant, G223S, has been reported in a child with a severe presentation of disease [16]. In the absence of further data, this variant was interpreted as a variant of unknown significance, however, as the patient’s phenotype was consistent with a diagnosis of B3GAT3-related disease, functional studies were pursued to further characterize the patient’s disease and novel variants.

To access the overall function of GlcAT-I, western blotting and expression analyses were performed using cultured primary fibroblasts from the patient and a healthy control. No difference in expression was detected in mRNA levels using two different primer pairs encompassing the first (Fragment A) and second (Fragment B) potential start codon (Fig. 1c, Additional file 1: Table S1). However western blotting demonstrated a significant decrease in protein levels (Fig. 1d). To examine glucuronyltransferase activity, assays of GlcAT-I activity were carried out using a cell lysate derived from the patient’s fibroblast cells. In stark comparison to control cells, no GlcAT-I activity was detected when lysate from the patient’s fibroblast cells was used as an enzyme source (Fig. 1e).

**Fig. 2:**
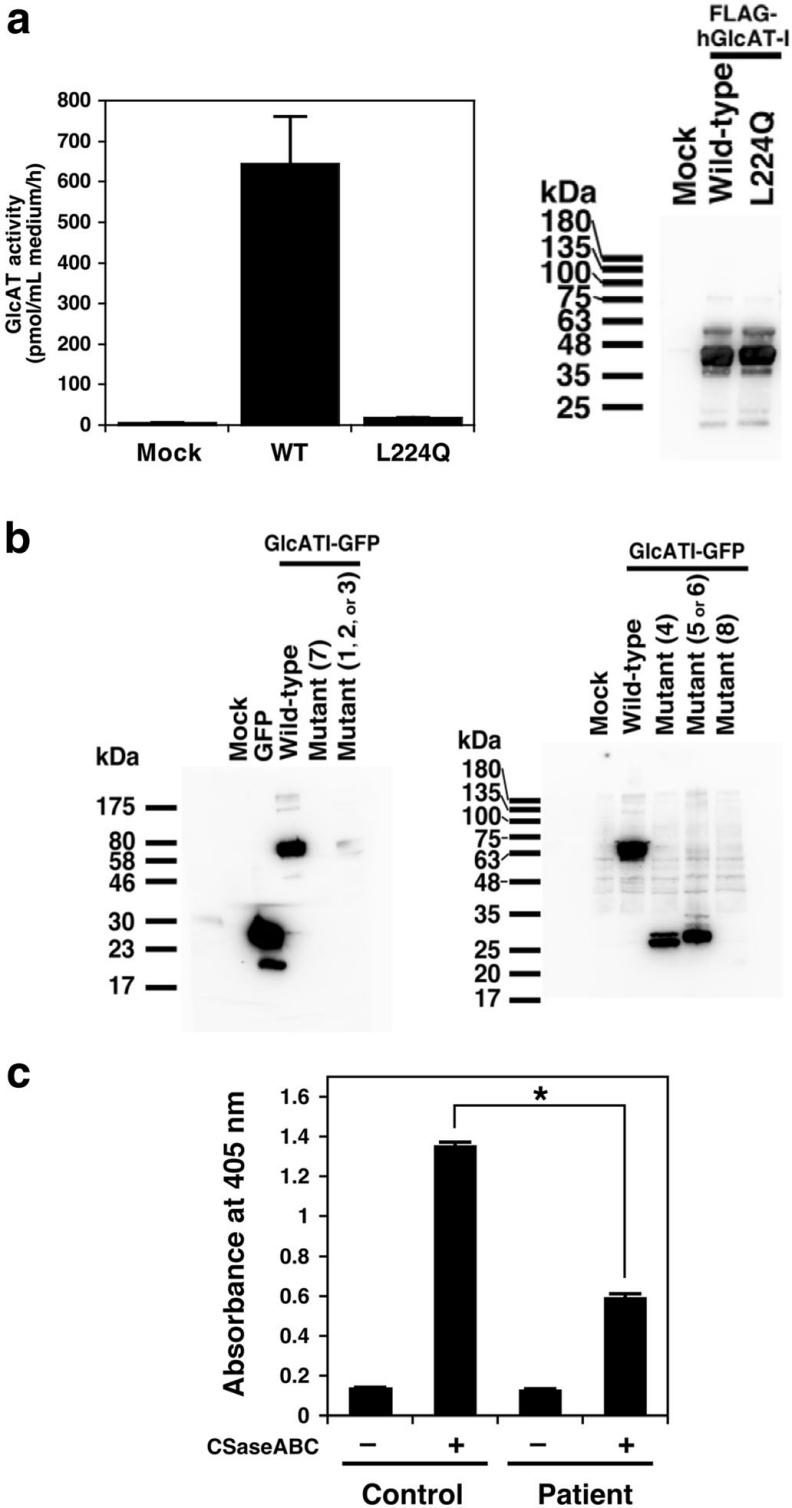
**a** Glucuronyltransferase activity of each enzyme protein was examined using the partially purified recombinant enzymes, UDP-[^14^C]-GlcA as the donor substrate, and Galβ1-3Galβ1-*O*-methyl as the acceptor substrate. Glucuronyltransferase activity was determined by incorporation of [^14^C]GlcA into the Galβ1-3Galβ1-*O*-methyl. “Mock” indicates the enzyme source from the HEK293T cells transfected with an empty vector. Values are the means ± SE (*n* = 3). *P < 0.0005 versus wild-type was calculated by the ANOVA Dunnett test. Two sets of the mutant enzyme, L224Q, were prepared from two separate conditioned media from the HEK293T cell cultures (*left*). The purified recombinant GlcAT-I separated by SDS-PAGE was detected with the horseradish peroxidase-conjugated anti-FLAG antibody (Wako). The broad signals of the FLAG-tagged GlcAT-I may indicate the *N*-glycosylated enzymes (~40 kDa). Western blotting analysis showed the expression of comparable amounts of these recombinant proteins. (*right*). **b** The GFP-tagged GlcAT-I and its mutant proteins separated by SDS-PAGE were detected with anti-GFP antibody and the horseradish peroxidase-conjugated anti-mouse IgG antibody as the primary and secondary antibodies, respectively. The deduced molecular masses of GFP-tagged wild-type GlcAT-I and the mutant proteins (mutants #1 ~ 8) were summarized in Additional file 1: Table S2. Although no obvious mutant proteins (No. 1 ~ 3, 7, and 8) of GlcAT-I were detected, GFP-tagged mutant proteins, #4 as well as #5 or 6, were clearly detected. Predicted molecular weights of the bands of #5 and #6 were 40.2 and 31.3 kDa, respectively (Additional file 1: Table S2). The GFP-tagged mutant protein #5 or 6 was detected at a molecular weight of ~ 30 kDa, suggesting that the observed GFP-tagged mutant protein might be mutant-#6 rather than mutant-#5. **c** Comparison of the amount of CS chains on the cell surface of fibroblasts from the patient and a healthy control by cell-based ELISA. *P < 0.0001

To further examine the effect of p.M1? and p.L224Q variants independently, wild-type and mutant vectors were constructed and glucuronyltransferase activity of the recombinant enzymes was tested. The GlcAT-I-L224Q construct showed a marked reduction to ~3% of the wild-type (Fig. 2a). However, protein levels were comparable to wild-type based on the western blotting (Fig. 2a). These results suggest that the L224Q variant affects the glucuronyltransferase activity and the biosynthesis of GAGs including chondroitin sulfate, dermatan sulfate, and heparan sulfate, and/or their proteoglycan forms.

To characterize the effects of the variant of the initiation codon on the encoded protein and to determine which alternative ATG may be utilized as the initiation codon in the open reading frame of the p.M1? gene, western blotting of C-terminally GFP-tagged mutant GlcAT-I proteins in all the possible reading frames (Additional file 1: Figure S4) was performed. Of note, there is not an in-frame alternative Met codon upstream of the Met1, therefore the c.1A > G variant is not predicted to result in an extended protein. Wild-type GlcAT-I was detected at a molecular weight of ~64 kDa (Fig. 2b). Although no obvious mutant proteins (No. 1 ~ 3, 5, 7, and 8) of GlcAT-I were detected, GFP-tagged mutant proteins, #4 as well as #6, were clearly detected (Fig. 2b). These results indicate that the third and fifth ATGs of the open reading frame, at the positions of c.233 and c.584 in mutant B3GAT3 might be utilized, respectively, to initiate translation (Additional file 1: Figure S3 and Additional file 1: Figure S4, Additional file 1: Table S2). The resultant proteins bear a sequence totally unrelated to the GlcAT-I enzyme, thus the allele (not the proteins) is considered functionally null.

After confirming the p.M1? variant was functionally null, to analyze how the reduced activity of GlcAT-I from the p.L224Q variant affects the biosynthesis of chondroitin sulfate (CS) side chains of proteoglycans, the relative numbers of CS chains were analyzed by cell-based ELISA with three kinds of anti-CS-stub antibodies using fibroblast cells from the patient and control. These antibodies can be used as probes for investigating the relative numbers of CS chains on the core proteins of proteoglycans [11]. CS-stub antibodies showed a markedly reduced binding to the patient cells compared to control subject (Fig. 2c), suggesting that the number of CS chains was significantly reduced in the fibroblast cells from the patient.

## Discussion

We have described the first patient of northern European descent with compound heterozygous variants in B3GAT3, expanding the phenotypic and genotypic spectrum associated with disease. Consistent with all patients reported to have B3GAT3-related disease, cmh000720 has a cardiac defects, joint laxity, dislocations, contractures, broad tips of fingers and a short neck [11, 15–17]. Our patient’s features overlap with those of a patient with severe disease associated with a homozygous G223S variant; including multiple fractures, arachnodactyly, and blue sclerae. He did not have a diaphragmatic hernia or small thorax, but does have restrictive lung disease requiring C-PAP/BiPAP at night. However, unlike other reported patients, our patient does not have short stature, currently his height is at the 13^th^%ile. Additionally, cmh000720 has macrocephaly, hypothryoidism and hypoglocemia, which have not been previously reported (Additional file 1: Table S3).

Independent in vitro studies demonstrated the p.M1? variant is functionally null and the p.L224Q variant has glucuronosyltransferase activity ~3% of wild-type. Both of cmh000720’s parents are healthy and no disease phenotype has been reported to date [11, 16, 17]. Consistent with the reported human phenotypes; null mice were embryonic lethal while heterozygous mice did not have a phenotype and were born at Mendelian frequencies [24]. Taken together, the data indicate that 50% glucuronosyltransferase activity compared to wild-type is sufficient for a normal phenotype. Further studies are needed to correlate the level of residual activity to resulting phenotype.

The cause of endocrine abnormalities in cmh000720 is not clear. Congenital hypothryoidism is relatively common, affecting ~1/2000 births. However, thyroid hormone has been shown to play a role in the expression of proteoglycans and so it cannot be ruled out that the patient’s B3GAT3 disease is the underlying cause [25]. Similarly, hypoglycemia has not been reported in other patients. Previous studies have demonstrated a decrease in chondroitin sulfate and heparan sulfate in patients with diabetes [26], therefore it is possible that the overall decrease in GAGs is contributing to his hypoglycemia.

## Conclusion

The linkeropathies are still a relatively new class of disorders, with the full disease spectrum yet to be defined. However, they should be considered as the differential diagnosis of patients with hypotonia, joint laxity, congenital heart disease and fractures. It is clear from the limited case reports to date that there is a wide spectrum of clinical severity associated with disease. As whole exome sequencing is quickly becoming the standard for clinical testing, these patients will likely be diagnosed earlier in their disease course. Further studies of additional patients are needed in order to fully elucidate the phenotypic spectrum and disease mechanisms of these disorders.

## Additional files

Additional file 1: Supplemental Data- Detailed Methodology. Detailed description of methods presented in the manuscript. **Figure S1.** Sanger Sequencing. Description and traces of sanger confirmation of *B3GAT3* variants. **Figure S2.** Computational Analysis of p.L224Q Variant. Computational modeling of wild-type and B3GAT3 p.L224Q variant, including amino acid conservation. **Figure S3.** Nucleotide and deduced amino acid sequences from the wild-type of the human GlcAT-I. Summary of potential alternative start codons within *B3GAT3*. **Figure S4.** Nucleotide and deduced amino acid sequences from the mutants of human GlcAT-I. Summary of putative variant proteins #1-8 in *B3GAT3*. **Table S1.** qPCR primers for GlATc-I mRNA Expression in Primary fibroblasts. Table of qPCR primer sequences. **Table S2.** The deduced molecular masses of the GFP-tagged wild-type GlcAT-I and the mutant proteins (mutants #1 ~ 8). Summary of predicted molecular weight of mutant proteins. **Table S3.** Primers for construction of the GFP-tagged hGlcAT-I expression vectors. PCR Primers for construction of vectors. **Table S4.** Phenotype Comparison of reported patients with B3GAT3 related disease. Phenotypic comparison of published patients with *B3GAT3*-related disease. (DOCX 736 kb)
