# Supplementary material for: Functional validation of novel compound heterozygous variants in B3GAT3 resulting in severe osteopenia and fractures: expanding the disease phenotype
Source: BMC Med Genet. 2016 Nov 21;17:86. doi: 10.1186/s12881-016-0344-9 (PMC5117547; doi:10.1186/s12881-016-0344-9)
Supplement: Additional file 1: — Supplemental Data- Detailed Methodology. Detailed description of methods presented in the manuscript. Figure S1. Sanger Sequencing. Description and traces of sanger confirmation of B3GAT3 variants. Figure S2. Computational Analysis of p.L224Q Variant. Computational modeling of wild-type and B3GAT3 p.L224Q variant, including amino acid conservation. Figure S3. Nucleotide and deduced amino acid sequences from the wild-type of the human GlcAT-I. Summary of potential alternative start codons within B3GAT3. Figure S4. Nucleotide and deduced amino acid sequences from the mutants of human GlcAT-I. Summary of putative variant proteins #1-8 in B3GAT3. Table S1. qPCR primers for GlATc-I mRNA Expression in Primary fibroblasts. Table of qPCR primer sequences. Table S2. The deduced molecular masses of the GFP-tagged wild-type GlcAT-I and the mutant proteins (mutants #1 ~ 8). Summary of predicted molecular weight of mutant proteins. Table S3. Primers for construction of the GFP-tagged hGlcAT-I expression vectors. PCR Primers for construction of vectors. Table S4. Phenotype Comparison of reported patients with B3GAT3 related disease. Phenotypic comparison of published patients with B3GAT3-related disease. (DOCX 736 kb) [file 12881_2016_344_MOESM1_ESM.docx]

**SUPPLEMENTAL DATA**

**Detailed Methodology**

*Whole Exome Sequencing*: Following informed consent, DNA was prepared for whole exome on the proband and his parents using the Nextera Rapid Capture Exome Kit (Illumina, San Diego, CA) according to manufacturer’s protocols. Samples were sequenced on an Illlumina HiSeq 2500 instrument with TruSeq v4 reagents, yielding paired end 125 nucleotide reads, with an average of 12.7 GB of data resulting in a mean 66X coverage. Alignment and variant calling were performed as previously reported [1]. Briefly, gapped alignment to reference sequences (GRCh37.p5) was performed with GSNAP and the GATK. Analysis was completed using custom-developed software, RUNES and VIKING [1, 2].

Initial analysis metrics applied were a hard filter of the variant being called in a minimum of 5 reads and a minor allele frequency <1% in an internal database resulting in 25 de novo variants and 29 variants recessive variants (compound heterozygous and homozygous). The same filtering criteria resulted in 3 variants for X-Linked inheritance and 2 for mitochondrial inheritance. Each variant was then assessed for pathogenicity using the proposed ACMG guidelines, which identified *B3GAT3* as the top candidate gene [3].

*Western blot analysis*: Primary fibroblasts of patient and sex- and age-matched control were cultivated in DMEM/HamsF12 (1:1) (Gibco) medium supplemented with 8% FCS (GE Healthcare), 2% Ultroser G (Cytogen GmbH) and 2 mM glutamine (PAA). Cells were seeded into six-well plates and allowed to grow to confluency. Cells were harvested by the addition of 500 µl Trypsin/EDTA (PAA) per well. After 5 min of incubation at 37°C cells were suspended in 5 ml cultivation medium and centrifuged (2 min, 2,000 rpm). Pellets of fibroblasts were washed twice with 2 ml PBS and lysed in 20 mM Tris (pH 8.0), 100 mM NaCl, 1 mM EDTA, 0.5 % Triton-X100, 1 mM PMSF, 1 mM Aprotinin, 50 µM MG132 and 1 mM DTT.

Afterwards BCA assay Kit (ThermoScientific) was performed according to the manufacturer´s protocol and 20 µg of each cell lysate was separated on a 10% SDS-PA gel (20 mA, 2 h). SDS-PA Gels were transferred onto polyvinylidene difluoride (PVDF) membranes and blotted 90 min with 1 mA / cm^2^. Membranes were blocked for 3 h at room temperature in PBS, 0.05% Tween-20 (PBST) and 5% (w/v) milk powder. Primary anti-B3GAT3 antibody (polyclonal mouse, Abnova) was diluted 1:500 in PBST, 0.5% (w/v) milk powder and used for membrane incubation over night at 4°C. Membrane was washed (3 x 10 min in PBST) and secondary horseradish peroxidase (HRP) coupled anti-mouse IgG (1:2,500) was applied for 1 h at room temperature. After membrane washing a ECL plus western blot detection Kit (Pierce) was used according to the manufacturers protocol.

As additional loading control experiment membranes were stripped in 0.2 M glycine, 3.5 mM SDS, 1% (v/v) Tween-20 (pH 2.2) for 20 min, washed in PBST and blocked for 1 h in PBST containing 5% (w/v) milk powder. Anti-actin antibody (1:5,000, polyclonal mouse; BD Bioscience) and anti-mouse IgG HRP-linked antibody (1:2,500) was used for detection.

All experiments were performed three times and a representative experiment is presented

*RNA extraction*: RNA was isolated from 25cm^2^ confluent primary fibroblast cultures (passages 4-6). 1 ml Trizol reagent (Ambion, Life Technologies) was added to pelleted cells and the samples were homogenized using gentleMACS TM Dissociator (MACS Miltenyi Biotec). Samples were centrifuged for 3 min at 3,000 rpm at 4°C. The supernatant was collected and incubated for 5 min at room temperature. 200 µl of chloroform was added and the samples were mixed rigorously. After 3 min of incubation the samples were centrifuged (15 min, 10,000 rpm, 4°C). The upper phase was mixed with 500 µl isopropanole and after 10 min of incubation the samples were centrifuged 10 min, 10,000rpm, 4°C. The pellet was washed twice with 500 µl 75% ethanol. The dried pellet was resuspended in 50 µl DEPC-water. Isolated RNA was subsequently used for cDNA synthesis.

*cDNA synthesis and quantitative PCR*: *B3GAT3* mRNA levels were analysed in primary fibroblasts by quantitative PCR (qPCR). 1 µg of total RNA was reverse transcribed into cDNA in a 20 µl reaction set up using random hexamer primers and ProtoScript® First Strand cDNA Synthesis Kit (NEB). A 20 µl qPCR reaction contained 10 ng cDNA, 1x HOT FIREPol® EvaGreen® qPCR Mix Plus (passive reference ROX, Solis Biodyne), and 1,25 µM primers. *B3GAT3* and *GAPDH* primer pair efficiencies were checked in a standard template dilution experiment and calculated to be similar (B3GAT3_1^st^_ATG: 94.9%; B3GAT3_2^nd^_ATG: 96.0%; GAPDH: 98.2%)(Primer sequences in table S1).

A qPCR run on StepOnePlus (ABI) consisted of an initial hot start for 15 min at 95°C, followed by 36 cycles with a denaturation step of 20s at 95°C, 30s annealing at 55°C and an extension step of 20s at 72°C. Afterwards, a melt curve was recorded. Therefore, the samples were heated for 30s at 95°C and then cooled for 30s at 65°C. The samples were then heated again to 95°C and in increments of 0.5°C fluorescence was measured to confirm homogeneity of the generated PCR product.

Relative expression was determined using the 2^−ΔΔCt^ method established by Livak and Schmittgen [4] and the SteponePlus software package (ABI). For the analyses of the *B3GAT3* mRNA levels in patient and control fibroblasts data were collected in triplicates and in three independent experiments (fibroblast passages 4-6) and given as mean +/-SD.

*GlcAT-I assay of the recombinant wild-type and mutant, L224Q, GlcAT-I proteins*: The expression vector of human GlcAT-I (wild-type), p3xFLAG-CMV8/hGlcAT-I, was used as described previously [5], and the mutant vector was constructed by overlapping extension PCR method [6]. Each vector was transiently transfected into HEK293T cells using the FuGENE HD DNA-transfection reagent (Promega, Madison, WI, USA). Three days after transfection, an aliquot of the conditioned media was individually incubated with an anti-FLAG affinity agarose resin (Wako, Osaka, Japan) at 4˚C for 4 h. To examine the expression of both recombinant enzymes, SDS-PAGE and western blotting were performed using an anti-FLAG antibody.

Glucuronyltransferase activity was examined as described previously [5]. Briefly, the enzyme-bound resin as an enzyme source, UDP-[^14^C]GlcA (Perkin Elmer, Boston, MA, USA) as the sugar donor substrate, and Galβ1-3Galβ1-*O*-methyl (Sigma, St. Louis, MO, USA) as the sugar acceptor were utilized for the assay. The reaction mixtures were incubated at 37 ˚C for 20 min. The radiolabeled products were separated from UDP-[^14^C]-GlcA using anion-exchange resin, AG 1-X8 (PO_4_^2-^ form), as described previously[7]. The isolated products, [^14^C]-GlcAβ1-3Galβ1-3Galβ1-*O*-methyl, were quantified in a liquid scintillation counter (LSC-7400, Hitachi-Aloka, Tokyo, Japan).

*Comparison of the GlcAT-I activities of fibroblast homogenates:* The homogenates of the fibroblasts were assayed using Galβ1-3Galβ1-*O*-methyl as an acceptor (220 nmol) and UDP-[^14^C]GlcA as a donor substrate, and then incubated for 4 h at 30 ˚C. The procedures thereafter were described above.

*Cell-based ELISA:*Cell-based ELISA was carried out using the protocol provided by R&D Systems Inc. (https://www.rndsystems.com/products/cell-based-elisas) with slightly modifications. Six unsaturated standard disaccharides derived from chondroitin sulfate (CS) and chondroitinase ABC (EC 4.2.2.20) from *Proteus vulgaris* were purchased from Seikagaku Biobusiness Corp. (Tokyo, Japan). Anti-CS-stub antibodies, 1B5, 2B6, and 3B3, were purchased from CosmoBio Co. (Tokyo, Japan). Briefly, fibroblasts from the patient and a control subject were cultured on 96-well plates (5,000 cells/well) for a day to determine the amount of CS. Cells were washed with phosphate-buffered saline (PBS) and treated with chondroitinase ABC at 37 ˚C for 30 min. After washing with PBS, the chondroitinase-treated cells were fixed with 4% paraformaldehyde, incubated with the primary antibodies, a mixture of anti-CS-stub antibodies (1B5, 2B6, and 3B3), and subsequently the cells were incubated with the secondary antibody, alkaline phosphatase-conjugated anti-mouse IgG. Then, the cells were incubated with the substrate, *p*-nitrophenyl phosphate, and analyzed by measuring the absorbance at 405 nm with an iMark microplate absorbance reader (Bio-Rad, Hercules, CA, USA).

*Construction of the pAcGFP-GlcAT-I vectors*: The expression vectors of the C-terminally GFP-tagged hGlcAT-I, pAcGFP-hGlcAT-I (wild-type and Met mutant) were constructed. Briefly, the expression vector of the full-length open reading frame encoding human GlcAT-I was amplified by PCR using pOTB7-human GlcAT-I (IMAGE clone #4299539, Open Biosystems, Huntsville, AL) as a template, KOD-Plus DNA polymerase (Toyobo, Tokyo, Japan), and the specific primers (5’- GG GGTACC GCCACC ATGAAGCTGAAGCTGA -3’ for sense primer of wild-type, 5’- GG GGTACC GCCACC *G*TGAAGCTGAAGCTGA -3’ for sense primer of Met mutant, and 5’- CG GAATTC CTTGGAAAACCACATCCT -3’ for antisense primer), where underlines and dot underline indicate the restriction enzyme site (KpnI and EcoRI) and Kozak sequence, respectively (Supplemental Table S3). The amplified fragments were digested with KpnI and EcoRI, and subcloned into pEF6/V5-His-A (Invitrogen). The resultant expression vector, pEF6/hGlcAT-I (wild-type and Met mutant)-V5-His, was digested with KpnI and EcoRV, and the cDNA fragments containing the open reading frame of GlcAT-I were inserted into the expression vector, pAcGFP-N1 (Clontech Laboratories, Inc.). It should be noted that the expression vectors, pAcGFP-hGlcAT-I (wild-type and Met mutant) do not contain 5’-UTR, and that the mutant vector was utilized to analyze the initiation of translation corresponding to the reading frame +1 (predicted mutant proteins #1-3 in Supplemental Fig. S4A).

The mutant expression vectors for analysis of the initiation codon(s) corresponding to the reading frame +2 and +3 (predicted mutant proteins #4-7 and #8 in Supplemental Figs. S4B and C, respectively) were also constructed. Each insert fragment was amplified by PCR using the mutant full-length vectors, pEF6-hGlcAT-I-V5/Met mutant #1-3 as the template, KOD-Plus DNA polymerase, and the specific primers (Supplemental Table S3). The amplified fragments were digested with KpnI and EcoRI, and subcloned into pEF6/V5-His-A. The resultant expression vector, pEF6/hGlcAT-I (Met mutants #4~8)-V5-His, was digested with KpnI and EcoRV, and the cDNA fragments were inserted into the expression vector, pAcGFP-N1 as described above.

*Characterization of the mutation in start codon of GlcAT-I*: The expression vector of the C-terminally GFP-tagged human GlcAT-I, pAcGFP-hGlcAT-I (wild-type), was constructed as described above. To examine the effects of mutations in the start codon of *GlcAT-I* on the expression of the encoded proteins, mutant vectors were also constructed in frame with C-terminal GFP tag. Each vector was transiently transfected into HEK293T cells using the FuGENE HD DNA-transfection reagent. Two days after transfection, the cultured cells were individually collected and the cell lysates were prepared using the mammalian-protein extraction reagent (ThermoFisher Scientific, Rockford, IL). To examine which ATG is utilized as the initiation codon, SDS-PAGE and western blotting were performed using an anti-GFP antibody, mFX75 (Wako, Japan).

**Supplemental Figure S1. Sanger Sequencing**

Sanger sequencing confirmed the *B3GAT3* variants identified through whole exome sequencing. The patient is heterozygous for the paternally inherited c.1A>G (p.Met1?) and maternally inherited c.671T>A (p.L224Q) variants.

**
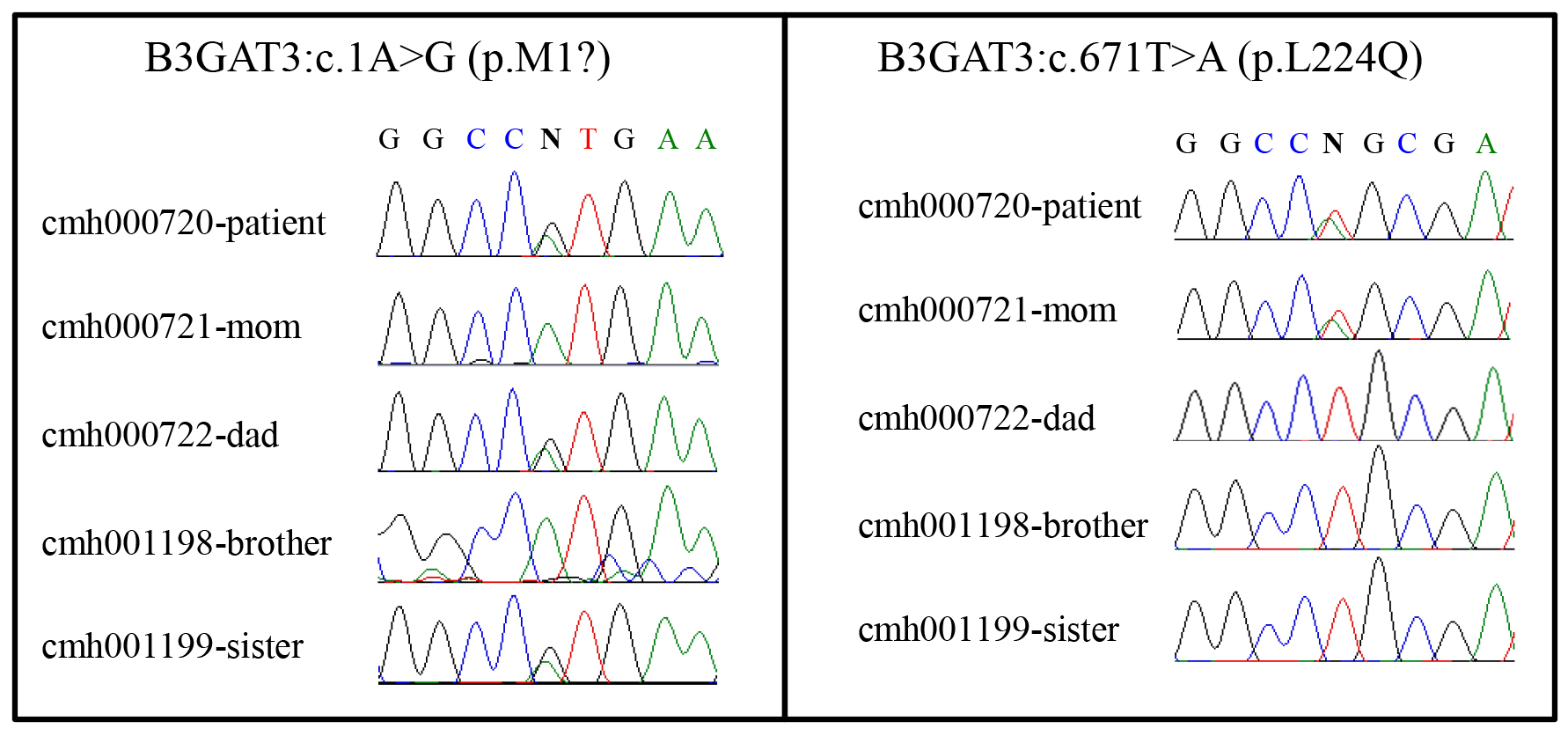
**

**Supplemental Figure S2. Computation Analysis of p. L224Q Variant**

Comparison of wild-type GlcAT-I (top, left) and mutant p.L224Q (top, right). GlcAT-I is encoded by the B3GAT3 gene and its active form is a dimer (monomers shown in yellow and light gray; secondary structures are shown as cartoon). Wildtype p.L224 is shown as orange and mutant p.224Q as brown spheres. The substrate UDP-glucuronic acid [UDP-GlcA] is displayed as dark gray ribbons. Additionally, the L224Q variant is predicted to have a destabilizing effect on the protein, with a score of -2.888Kcal/mol in Chain A. (structures were modeled using pdb-file 1KWS and DUET)[8].

The position 224 is also conserved across species (bottom).

**
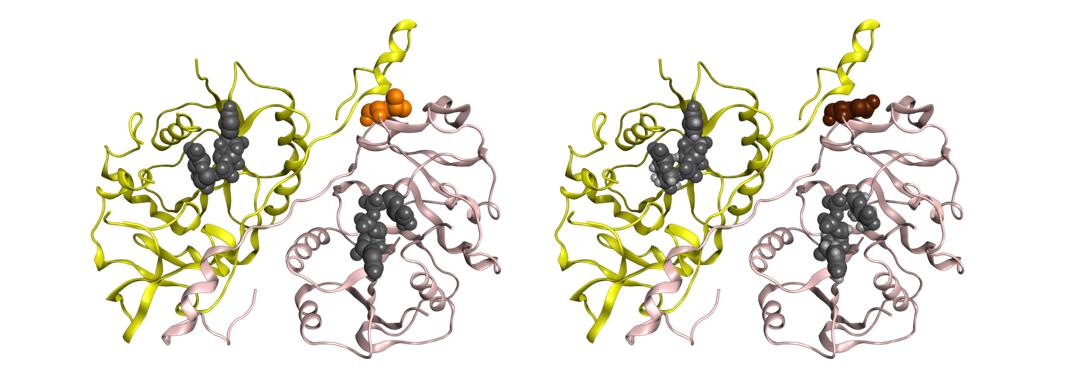
**

**
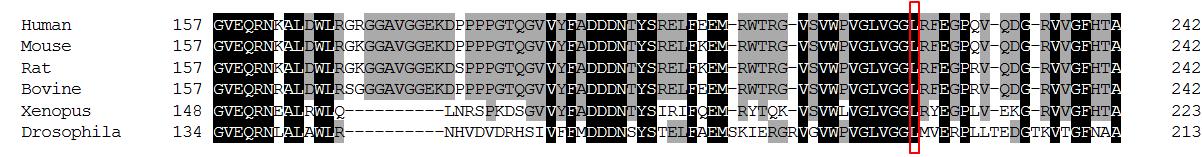
**

**Supplemental Figure S3. Nucleotide and deduced amino acid sequences from the wild-type of the human GlcAT-I.**

The green box and the asterisk indicate the initiation and stop codons, respectively. The yellow boxes represent the ATG but not initiation codon in *B3GAT3*.

1 atgaagctgaagctgaagaacgtgtttctcgcctacttcctggtgtcgatcgccggcctcctctacgcgctggtacagctcggccagcca 90

M K L K L K N V F L A Y F L V S I A G L L Y A L V Q L G Q P

91 tgtgactgccttcctcccctgcgggcagcagccgagcagctacggcagaaggatctgaggatttcccagctgcaagcggaactccgacgg 180

C D C L P P L R A A A E Q L R Q K D L R I S Q L Q A E L R R

181 ccaccccctgcccctgcccagccccctgaacccgaggccctgcctactatctatgttgttacccccacctatgccaggctggtacagaag 270

P P P A P A Q P P E P E A L P T I Y V V T P T Y A R L V Q K

271 gcagagctggtacgactgtcccagacactgagcctggtgccccggctgcattggctgctggtggaggatgctgagggtcccaccccgctg 360

A E L V R L S Q T L S L V P R L H W L L V E D A E G P T P L

361 gtctcagggctgctggctgcctctggcctcctcttcacacacctggtggtcctcacgcccaaagcccagcggcttcgggagggcgagcct 450

V S G L L A A S G L L F T H L V V L T P K A Q R L R E G E P

451 ggctgggttcatccccgtggtgtcgagcagcggaacaaggccctggactggctccggggcagagggggtgctgtgggtggggagaaggac 540

G W V H P R G V E Q R N K A L D W L R G R G G A V G G E K D

541 ccaccaccaccagggacccaaggagtcgtctactttgctgacgatgacaacacctacagccgggagctgtttgaggagatgcgctggacc 630

P P P P G T Q G V V Y F A D D D N T Y S R E L F E E M R W T

631 cgtggtgtctcagtgtggcctgtggggctggtgggcggcctgcgattcgagggccctcaggtacaggacggccgggtagtgggcttccac 720

R G V S V W P V G L V G G L R F E G P Q V Q D G R V V G F H

721 acagcatgggagcccagcaggcccttccctgtggatatggctggatttgccgtggccctgcccttgctgttagataagcccaatgcccaa 810

T A W E P S R P F P V D M A G F A V A L P L L L D K P N A Q

811 tttgattccaccgctccccggggccacctggagagcagtcttctgagccaccttgtggatcccaaggacctggagccacgggctgccaac 900

F D S T A P R G H L E S S L L S H L V D P K D L E P R A A N

901 tgcactcgggtactggtgtggcatactcggacagagaagcccaagatgaagcaggaggagcagctgcagcggcagggccggggctcagac 990

C T R V L V W H T R T E K P K M K Q E E Q L Q R Q G R G S D

991 ccagcaattgaggtgtga 1008

P A I E V *

**Supplemental Figure S4. Nucleotide and deduced amino acid sequences from the mutants of human GlcAT-I**

The mutation, **A**TG –> **G**TG, in the initiation codon of human GlcAT-I may affect the translation initiation. The deduced amino acid sequences of the putative mutant proteins (#1-8) are described below. Magenta and yellow boxes represent the position at the mutation of the first ATG to GTG and the other putative translation initiation codons, respectively.

**A) frame: +1 (mutant proteins #1-3)**

1 Gtgaagctgaagctgaagaacgtgtttctcgcctacttcctggtgtcgatcgccggcctcctctacgcgctggtacagctcggccagcca 90

91 tgtgactgccttcctcccctgcgggcagcagccgagcagctacggcagaaggatctgaggatttcccagctgcaagcggaactccgacgg 180

181 ccaccccctgcccctgcccagccccctgaacccgaggccctgcctactatctatgttgttacccccacctatgccaggctggtacagaag 270

271 gcagagctggtacgactgtcccagacactgagcctggtgccccggctgcattggctgctggtggaggatgctgagggtcccaccccgctg 360

361 gtctcagggctgctggctgcctctggcctcctcttcacacacctggtggtcctcacgcccaaagcccagcggcttcgggagggcgagcct 450

451 ggctgggttcatccccgtggtgtcgagcagcggaacaaggccctggactggctccggggcagagggggtgctgtgggtggggagaaggac 540

541 ccaccaccaccagggacccaaggagtcgtctactttgctgacgatgacaacacctacagccgggagctgtttgaggagatgcgctggacc 630

M R W T

631 cgtggtgtctcagtgtggcctgtggggctggtgggcggcctgcgattcgagggccctcaggtacaggacggccgggtagtgggcttccac 720

R G V S V W P V G L V G G L R F E G P Q V Q D G R V V G F H

721 acagcatgggagcccagcaggcccttccctgtggatatggctggatttgccgtggccctgcccttgctgttagataagcccaatgcccaa 810

T A W E P S R P F P V D M A G F A V A L P L L L D K P N A Q

811 tttgattccaccgctccccggggccacctggagagcagtcttctgagccaccttgtggatcccaaggacctggagccacgggctgccaac 900

F D S T A P R G H L E S S L L S H L V D P K D L E P R A A N

901 tgcactcgggtactggtgtggcatactcggacagagaagcccaagatgaagcaggaggagcagctgcagcggcagggccggggctcagac 990

C T R V L V W H T R T E K P K M K Q E E Q L Q R Q G R G S D

991 ccagcaattgaggtgtga 1008

P A I E V *

**B) frame: +2 (mutant proteins #4-7)**

1 Gtgaagctgaagctgaagaacgtgtttctcgcctacttcctggtgtcgatcgccggcctcctctacgcgctggtacagctcggccagcca 90

91 tgtgactgccttcctcccctgcgggcagcagccgagcagctacggcagaaggatctgaggatttcccagctgcaagcggaactccgacgg 180

181 ccaccccctgcccctgcccagccccctgaacccgaggccctgcctactatctatgttgttacccccacctatgccaggctggtacagaag 270

M L L P P P M P G W Y R R

271 gcagagctggtacgactgtcccagacactgagcctggtgccccggctgcattggctgctggtggaggatgctgagggtcccaccccgctg 360

Q S W Y D C P R H * M L R V P P R W

361 gtctcagggctgctggctgcctctggcctcctcttcacacacctggtggtcctcacgcccaaagcccagcggcttcgggagggcgagcct 450

S Q G C W L P L A S S S H T W W S S R P K P S G F G R A S L

451 ggctgggttcatccccgtggtgtcgagcagcggaacaaggccctggactggctccggggcagagggggtgctgtgggtggggagaaggac 540

A G F I P V V S S S G T R P W T G S G A E G V L W V G R R T

541 ccaccaccaccagggacccaaggagtcgtctactttgctgacgatgacaacacctacagccgggagctgtttgaggagatgcgctggacc 630

H H H Q G P K E S S T L L T M T T P T A G S C L R R C A G P

631 cgtggtgtctcagtgtggcctgtggggctggtgggcggcctgcgattcgagggccctcaggtacaggacggccgggtagtgggcttccac 720

V V S Q C G L W G W W A A C D S R A L R Y R T A G *

721 acagcatgggagcccagcaggcccttccctgtggatatggctggatttgccgtggccctgcccttgctgttagataagcccaatgcccaa 810

M P N

811 tttgattccaccgctccccggggccacctggagagcagtcttctgagccaccttgtggatcccaaggacctggagccacgggctgccaac 900

L I P P L P G A T W R A V F *

901 tgcactcgggtactggtgtggcatactcggacagagaagcccaagatgaagcaggaggagcagctgcagcggcagggccggggctcagac 990

991 ccagcaattgaggtgtga 1008

**C) frame: +3 (mutant protein #8)**

1 Gtgaagctgaagctgaagaacgtgtttctcgcctacttcctggtgtcgatcgccggcctcctctacgcgctggtacagctcggccagcca 90

M

91 tgtgactgccttcctcccctgcgggcagcagccgagcagctacggcagaaggatctgaggatttcccagctgcaagcggaactccgacgg 180

*

181 ccaccccctgcccctgcccagccccctgaacccgaggccctgcctactatctatgttgttacccccacctatgccaggctggtacagaag 270

271 gcagagctggtacgactgtcccagacactgagcctggtgccccggctgcattggctgctggtggaggatgctgagggtcccaccccgctg 360

361 gtctcagggctgctggctgcctctggcctcctcttcacacacctggtggtcctcacgcccaaagcccagcggcttcgggagggcgagcct 450

451 ggctgggttcatccccgtggtgtcgagcagcggaacaaggccctggactggctccggggcagagggggtgctgtgggtggggagaaggac 540

541 ccaccaccaccagggacccaaggagtcgtctactttgctgacgatgacaacacctacagccgggagctgtttgaggagatgcgctggacc 630

631 cgtggtgtctcagtgtggcctgtggggctggtgggcggcctgcgattcgagggccctcaggtacaggacggccgggtagtgggcttccac 720

721 acagcatgggagcccagcaggcccttccctgtggatatggctggatttgccgtggccctgcccttgctgttagataagcccaatgcccaa 810

M G A Q Q A L P C G Y G W I C R G P A L A V R *

811 tttgattccaccgctccccggggccacctggagagcagtcttctgagccaccttgtggatcccaaggacctggagccacgggctgccaac 900

901 tgcactcgggtactggtgtggcatactcggacagagaagcccaagatgaagcaggaggagcagctgcagcggcagggccggggctcagac 990

991 ccagcaattgaggtgtga 1008

**Supplemental Table S1. qPCR primers for GlATc-I mRNA Expression in Primary fibroblasts.**

Two sets of intron spanning qPCR primers were devised to test the expression levels of *B3GAT3* mRNA in primary fibroblasts from cmh000720 and an age matched control. The first primer set (Fragment A) is before the potential second start codon and has a fragment size of 84bp. The second primer set (Fragment B) results in a 92bp fragment size and is after the second potential start codon. GAPDH was used as an endogenous control (87bp fragment size).

**Supplemental Table S2. The deduced molecular masses of the GFP-tagged wild-type GlcAT-I and the mutant proteins (mutants #1~8).**

| Proteins | Predicted molecular weight  of the mutant proteins (kDa) | Predicted molecular weight  as the GFP-fusion proteins (kDa) |
| --- | --- | --- |
| **GlcAT-I wild-type** | 37.1 | **64.0** |
| **Mutant-1** | 14.4 | **41.3** |
| **Mutant-2** | 9.2 | **36.1** |
| **Mutant-3** | 2.3 | **29.2** |
| **Mutant-4** | 2.8 | **29.7** |
| **Mutant-5** | 13.3 | **40.2** |
| **Mutant-6** | 4.4 | **31.3** |
| **Mutant-7** | 1.9 | **28.8** |
| **Mutant-8** | 2.4 | **29.3** |

The molecular weight of the recombinant GFP protein was 26.9 kDa.

**Supplemental Table S3. Primers for construction of the GFP-tagged hGlcAT-I expression vectors.**

The single and dot underlines indicate recognition sequences by restriction enzymes, KpnI or EcoRI, and Kozak sequence, respectively. The italic “*G*” of the primer sequence represents the mutation position from “A” in the patient.

| pAcGFP-N1-  hGlcAT-I | Sense primer (5’–3’) | Antisense primer (5’–3’) |
| --- | --- | --- |
| **wild-type** | GG GGTACC GCCACC ATGAAGCTGAAGCTGA | CG GAATTC CACCTCAATTGCTGGGTC |
| **Mutants #1-3** | GG GGTACC GCCACC *G*TGAAGCTGAAGCTGA |  |
| **Mutant #4** | TAATACGACTCACTATAGGG  (T7 primer) | CG GAATTC GTGTCTGGGACAGTCGTA |
| **Mutants #5, 6** |  | CG GAATTC CCCGGCCGTCCTGTACCT |
| **Mutant #7** |  | CG GAATTC GAAGACTGCTCTCCAGGT |
| **Mutant #8** |  | CG GAATTC TCTAACAGCAAGGGCAGG |

**Supplemental Table S4: Phenotype Comparison of reported patients with B3GAT3 related disease**[5, 9, 10]

**Supplemental References**

1. Saunders CJ, Miller NA, Soden SE, Dinwiddie DL, Noll A, Alnadi NA, Andraws N, Patterson ML, Krivohlavek LA, Fellis J *et al*: Rapid whole-genome sequencing for genetic disease diagnosis in neonatal intensive care units. *Sci Transl Med* 2012, 4(154):154ra135.

2. Soden SE, Saunders CJ, Willig LK, Farrow EG, Smith LD, Petrikin JE, LePichon JB, Miller NA, Thiffault I, Dinwiddie DL *et al*: Effectiveness of exome and genome sequencing guided by acuity of illness for diagnosis of neurodevelopmental disorders. *Sci Transl Med* 2014, 6(265):265ra168.

3. Richards S, Aziz N, Bale S, Bick D, Das S, Gastier-Foster J, Grody WW, Hegde M, Lyon E, Spector E *et al*: Standards and guidelines for the interpretation of sequence variants: a joint consensus recommendation of the American College of Medical Genetics and Genomics and the Association for Molecular Pathology. *Genet Med* 2015, 17(5):405-424.

4. Livak KJ, Schmittgen TD: Analysis of relative gene expression data using real-time quantitative PCR and the 2(-Delta Delta C(T)) Method. *Methods* 2001, 25(4):402-408.

5. Baasanjav S, Al-Gazali L, Hashiguchi T, Mizumoto S, Fischer B, Horn D, Seelow D, Ali BR, Aziz SA, Langer R *et al*: Faulty initiation of proteoglycan synthesis causes cardiac and joint defects. *Am J Hum Genet* 2011, 89(1):15-27.

6. Zhao S, Yamamoto R: Detection of Mycoplasma meleagridis by polymerase chain reaction. *Vet Microbiol* 1993, 36(1-2):91-97.

7. Tone Y, Kitagawa H, Imiya K, Oka S, Kawasaki T, Sugahara K: Characterization of recombinant human glucuronyltransferase I involved in the biosynthesis of the glycosaminoglycan-protein linkage region of proteoglycans. *FEBS Lett* 1999, 459(3):415-420.

8. Pires DE, Ascher DB, Blundell TL: DUET: a server for predicting effects of mutations on protein stability using an integrated computational approach. *Nucleic Acids Res* 2014, 42(Web Server issue):W314-319.

9. Jones KL, Schwarze U, Adam MP, Byers PH, Mefford HC: A homozygous B3GAT3 mutation causes a severe syndrome with multiple fractures, expanding the phenotype of linkeropathy syndromes. *Am J Med Genet A* 2015, 167A(11):2691-2696.

10. von Oettingen JE, Tan WH, Dauber A: Skeletal dysplasia, global developmental delay, and multiple congenital anomalies in a 5-year-old boy-report of the second family with B3GAT3 mutation and expansion of the phenotype. *Am J Med Genet A* 2014, 164A(6):1580-1586.
